# Supplementary material for: Elucidation of divergent desaturation pathways in the formation of vinyl isonitrile and isocyanoacrylate
Source: Nat Commun. 2022 Sep 12;13:5343. doi: 10.1038/s41467-022-32870-4 (PMC9467999; doi:10.1038/s41467-022-32870-4)
Supplement: Supplementary file 1 — Supplementary Information [file 41467_2022_32870_MOESM1_ESM.pdf]

Supplementary Information

Elucidation of divergent desaturation pathways in the formation of vinyl isonitrile and isocyanoacrylate

Wantae Kim,[1,†] Tzu-Yu Chen,[2,†] Lide Cha,[2] Grace Zhou,[3] Kristi Xing,[3] Nicholas Koenig Canty,[2] Yan Zhang [3,4,*] and Wei-chen Chang[2,*]

[1,] McKetta Department of Chemical Engineering, [3] Department of Molecular Biosciences and [4] Institute for Cellular and Molecular Biology, University of Texas, Austin, Texas, U.S.A.

[2] Department of Chemistry, NC State University, North Carolina, U.S.A.

[†]These authors contribute equally to the work.[*] Corresponding should be addressed to Yan Zhang (jzhang@cm.utexas.edu) or Wei-chen Chang (wchang6@ncsu.edu).

Contents:

1. Supplementary methods……………………………………………………………………P2

1.1 General chemical procedure ………………………………………… P2

1.2 Preparation of [2-^{13}C]-**6**, **6**, **9** and **10** ………………………………… P2

1.3 Cloning, protein expression and purification of PIsnB, PvcB and PIsnB-Ah …… P4

1.4 *In vitro* assays of PvcB, PIsnB and PIsnB-Ah catalyzed reactions ……………… P6

1.5 Using ^{13}C-NMR spectroscopy to follow enzymatic reactions ……………………… P8

1.6 Differential scanning fluorimetry assay to measure protein stability……………… P8

1.7 Crystallization and X-ray crystallography ………………………………… P8

1.8 Sequence alignment and analysis…………………………………………… P9

2. Supplementary Figures 1-23 …………………………………………………… P10

3. Supplementary Table ………………………………………………………… P27

4. Supplementary References …………………………………………………… P28

1. Supplementary Methods

1.1 General chemical procedures

The chemical shift values are reported in δ values (parts per million, ppm) relative to the standard chemical shift for the proton residue peak and ^{13}C peak in the deuterated solvent, $CDCl_3$, D_2O or CD_3OD.[1] The coupling constant (*J*) values are expressed in hertz (Hz). Thin-layer chromatography (TLC) was performed on silica gel plates. Compounds on TLC were visualized by illumination under UV light (254 nm), dipped into $KMnO_4$ solution followed by charring on a hot plate. Solvent systems are expressed as a percentage of the more polar component with respect to total volume (v/v %). Silica gel (230-400 mesh) was used for flash column chromatography. Evaporations were carried out under reduced pressure (water aspirator or vacuum pump) with the bath temperature below 50°C unless specified otherwise. Materials obtained from commercial suppliers were used directly without further purification.

1.2 Preparation of [2-^{13}C]-**6**, **6**, **9** and **10**

Preparation of [2-^{13}C]-**6** and **6**

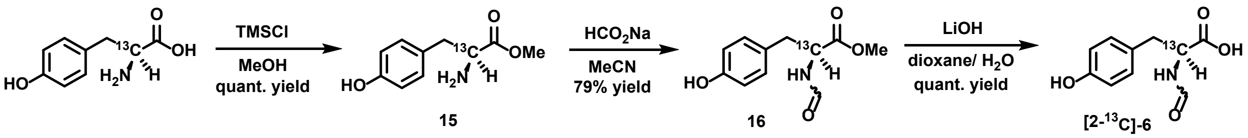

Adapted from the reported literature[2], [2-^{13}C]-**6** was prepared. To a solution of L-tyrosine-2-^{13}C (250.0 mg, 1.37 mmol) in methanol (4.6 mL) was added trimethylsilyl chloride (TMSCl, 0.7 mL, 5.49 mmol) at 0°C. After addition, the mixture was kept stirring at room temperature overnight. Subsequently, the solvent was removed under reduced pressure to give compound **15** without further purification (quant. yield). ^{1}H NMR (500 MHz, D_2O) δ 7.18 (d, *J* = 8.0 Hz, 2H), 6.92 (d, *J* = 8.5 Hz, 2H), 4.22-4.57 (dm, $J_{C\text{-}H}$ = 149.0 Hz, 1H), 3.86 (s, 3H), 3.25-3.31 (m, 1H), 3.15-3.22 (m, 1H); ^{13}C NMR (125 MHz, D_2O) δ 54.1.

To a suspension of compound **15** (1.37 mmol) in acetonitrile (4.6 mL) was added sodium formate (HCO_2Na, 186.6 mg, 2.74 mmol) at room temperature. Subsequently, the reaction was brought to reflux until no starting material was detected by TLC. After cooling down to room temperature, the crude product was concentrated under reduced pressure and purified by silica gel column chromatography (pure ethyl acetate) to give compound **16** (244.2 mg, 79% yield). Compound **16** presents as two tautomers in a ratio of 1:0.13.

Major tautomer: ^{1}H NMR (500 MHz, CD_3OD) δ 8.01 (d, *J* = 5.5 Hz, 1H), 7.00 (d, *J* = 8.5 Hz, 2H), 6.70 (d, *J* = 8.5 Hz, 2H), 4.52-4.90 (ddd, *J* = 5.5 and 7.5 Hz, $J_{C\text{-}H}$ = 143.5 Hz, 1H), 3.70 (s, 3H), 3.01-3.08 (m, 1H), 2.86-2.93 (m, 1H); ^{13}C NMR (125 MHz, CD_3OD) δ 54.0.

Minor tautomer: ^{1}H NMR (500 MHz, CD_3OD) δ 7.66 (d, *J* = 1.5 Hz, 1H), 7.02 (d, *J* = 8.5 Hz, 2H), 6.72 (d, *J* = 8.5 Hz, 2H), 4.20-4.50 (ddd, *J* = 4.5 and 10.0 Hz, $J_{C\text{-}H}$ = 140.5 Hz,

1H), 3.75 (s, 3H), 3.10-3.17 (m, 1H), 2.76-2.83 (m, 1H); ^{13}C NMR (125 MHz, CD_3OD) δ 58.4.

To a solution of compound **16** (244.2 mg, 1.1 mmol) in 1,4-dioxane (5.5 mL) and water (4.2 mL) was added lithium hydroxide solution (LiOH, 1M in H_2O, 1.2 mL, 1.2 mmol) at room temperature. The mixture was kept stirring at room temperature until no starting material was detected by TLC. After solvent removal, [2-^{13}C]-**6** was obtained in its lithium salt form (quant. yield). [2-^{13}C]-**6** presents as two tautomers in a ratio of 1:0.15.

Major tautomer: ^{1}H NMR (500 MHz, D_2O) δ 7.87 (d, *J* = 5.0 Hz, 1H), 6.92 (d, *J* = 8.5 Hz, 2H), 6.52 (d, *J* = 8.5 Hz, 2H), 4.17-4.51 (ddd, *J* = 5.0 and 8.0 Hz, $J_{C\text{-}H}$ = 142.0 Hz, 1H), 2.90-2.96 (m, 1H), 2.71-2.79 (m, 1H); ^{13}C NMR (125 MHz, D_2O) δ 177.9, 163.5, 130.4, 123.3, 118.3, 55.3, 36.3.

Minor tautomer: ^{1}H NMR (500 MHz, D_2O) δ 7.40 (d, *J* = 1.5 Hz, 1H), 6.90 (d, *J* = 7.0 Hz, 2H), 6.53 (d, *J* = 7.0 Hz, 2H), 3.82-4.14 (ddd, *J* = 4.0 and 9.5 Hz, $J_{C\text{-}H}$ = 140.0 Hz, 1H), 2.96-3.01 (m, 1H), 2.61-2.68 (m, 1H); ^{13}C NMR (125 MHz, D_2O) δ 178.3, 163.7, 130.8, 123.3, 118.4, 59.7, 36.6.

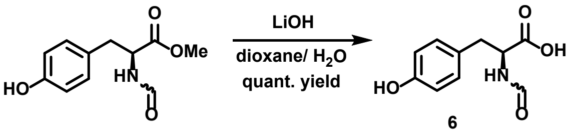

Compound **6** was prepared in an analogous manner as of [2-^{13}C]-**6** preparation. Compound **6** presents as two tautomers in a ratio of 1:0.15.

Major tautomer: ^{1}H NMR (500 MHz, D_2O) δ 7.85 (s, 1H), 6.92 (d, *J* = 8.0 Hz, 2H), 6.52 (d, *J* = 7.5 Hz, 2H), 4.33 (dd, *J* = 5.0 and 8.0 Hz, 1H), 2.93 (dd, *J* = 5.0 and 14.0 Hz, 1H), 2.75 (dd, *J* = 8.0 and 14.5 Hz, 1H); ^{13}C NMR (125 MHz, D_2O) δ 178.1, 163.5, 130.4, 123.5, 118.1, 55.3, 36.5.

Minor tautomer: ^{1}H NMR (500 MHz, D_2O) δ 7.37 (s, 1H), 6.89 (d, *J* = 8.0 Hz, 2H), 6.54 (d, *J* = 7.5 Hz, 2H), 3.96 (dd, *J* = 4.5 and 9.5 Hz, 1H), 2.98 (dd, *J* = 4.0 and 14.0 Hz, 1H), 2.64 (dd, *J* = 9.0 and 14.0 Hz, 1H); ^{13}C NMR (125 MHz, D_2O) δ 166.6, 163.2, 130.8, 123.4, 118.2, 59.7, 38.4.

<u>Preparation of **9** and **10**</u>

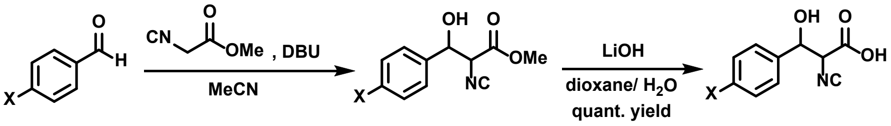

Adapted from the reported literature,[2] **9** and **10** were prepared. To a solution of methyl 2-isocyanoacetate (254.6 mg, 2.57 mmol) in acetonitrile (MeCN, 14 mL) was added 1,8-diazabicyclo[5.4.0]undec-7-ene (DBU, 0.38 mL, 2.57 mmol) at room temperature. After

the mixture was stirred for 1 hour, a solution of benzaldehyde (0.30 mL, 2.92 mmol) in acetonitrile (MeCN, 14 mmol) was added into the mixture dropwise at room temperature. Subsequently, the reaction was kept stirring at same temperature until no starting material was detected by TLC. The crude product was concentrated under reduced pressure and purified by silica gel column chromatography (*n*-hexanes/ ethyl acetate = 1/1 (v/v)) to give compound **17** (55.9 mg, 11% yield). ^{1}H NMR (500 MHz, $CDCl_3$) δ 7.59-7.46 (m, 5H), 5.85 (d, J = 7.5 Hz, 1H), 4.79 (d, J = 8.0 Hz, 1H), 4.00 (s, 3H); ^{13}C NMR (125 MHz, $CDCl_3$) δ 170.9, 156.4, 138.9, 129.0, 128.8, 125.6, 82.2, 75.3, 52.9.

To a solution of **17** (55.9 mg, 0.27 mmol) in 1,4-dioxane (1.4 mL) was added lithium hydroxide solution (LiOH, 1M in H_2O, 0.27 mL, 0.27 mmol) followed by water (1.4 mL) at room temperature. The mixture was kept stirring at same temperature until no starting material was detected by TLC. After solvent removal, **9** was obtained in its lithium salt form (quant. yield) along with the corresponding formyl compound with the ratio of ~1:0.1. ^{1}H NMR (500 MHz, D_2O) δ 7.48-7.33 (m, 5H), 5.54 (d, J = 7.5 Hz, 1H), 4.42 (d, J = 7.5 Hz, 1H); ^{13}C NMR (125 MHz, D_2O) δ 177.8, 157.1, 139.2, 129.0, 128.9, 125.8, 84.2, 76.5.

Compound **18** (51.3 mg, 8.1% yield) was prepared in an analogous manner as of **17** by using 4-fluorobenzaldehyde as a starting material. ^{1}H NMR (500 MHz, $CDCl_3$) δ 7.51-7.45 (m, 2H), 7.28-7.24 (m, 2H), 5.84 (d, J = 8.0 Hz, 1H), 4.77 (d, J = 8.0 Hz, 1H), 4.01 (s, 3H); ^{13}C NMR (125 MHz, $CDCl_3$) δ 170.8, 162.9 (d, $J_{F\text{-}C}$ = 246.2 Hz), 156.3, 134.8 (d, $J_{F\text{-}C}$ = 3.2 Hz), 127.5 (d, $J_{F\text{-}C}$ = 8.3 Hz), 116.0 (d, $J_{F\text{-}C}$ = 21.6 Hz), 81.6, 75.3, 53.0.

Compound **10** (quant. yield) was obtained along with the corresponding formyl compound with the ratio of ~1:0.1. ^{1}H NMR (500 MHz, D_2O) δ 7.43-7.35 (m, 2H), 7.20-7.14 (m, 2H), 5.53 (d, J = 7.5 Hz, 1H), 4.41 (d, J = 7.5 Hz, 1H); ^{13}C NMR (125 MHz, D_2O) δ 177.7, 162.6 (d, $J_{F\text{-}C}$ = 243.7 Hz), 157.0, 135.1 (d, $J_{F\text{-}C}$ = 3.1Hz), 128.0 (d, $J_{F\text{-}C}$ = 8.6 Hz), 115.7 (d, $J_{F\text{-}C}$ = 21.6 Hz), 83.6, 76.4; ^{19}F NMR (564 MHz, D_2O) δ -113.7.

Compounds **3**, **4**, **5**, **7**, **13** and **14** were prepared following the literature procedure.[2]

1.3 Cloning, protein expression and purification of PlsnB, PvcB and PlsnB-Ah

The DNA sequences encoding PlsnB, PvcB and PlsnB-Ah from *Photorhabdus luminescens* (NCBI Reference Sequence: WP_011147037.1), *Pseudomonas aeruginosa* PAO1 (NCBI Reference Sequence: AAC21672.1) and *Aeromonas hydrophila* (NCBI Reference Sequence: WP_017765143.1) were codon-optimized for overexpression in *E. coli*, synthesized, and inserted into expression vector pET-28a between the NdeI and XhoI sites with a 6xHis tag attached to the N-terminus. The codon-optimized gene sequences are shown below.

pisnB

ATGGGCAGCAGCCATCATCATCATCATCACAGCAGCGGCCTGGTGCCGCGCGGC
AGCCATATGATGACCGAACTGAATAGCTTTCAGACCGAAGAAATTACCCCGTTTGG
TCTGAAAATTACACCGCAGTATAGCGATCAGCATATTGATACCCTGCCGGTTGAAC
AGCTGAAAGAACTGGCACGTAAACATCATCTGCTGATTCTGCGTGGTTTTAAAAGC
GATCTGAGCGATCATGAGAAATATGAGAAATACGCACGTAACTGGGGTGAAATTAT
GATGTGGCCGTTTGGTGCAATTCTGGATGTTCGTGAACATCAGGATGCAACCGATC
ATGTTTTTGATAATAGCTATATGCCGCTGCATTGGGATGGTATGTATAAACCGACCA
TCCCGGAATTTATCATGTTTCATTGTGCACATGCACCGGAAAGCGATCAGGGTGGT
CGTACCACCTTTGTTAATACCCGTCGTGTTGTTGCAAATGCAACCCAGCAGCAGCT
GGAACAGTGGAAAAACATTAGCATTACCTACCGCATCAACAAAGTGACCCATTATG
GTGGTGAAGTTCATAGTCCGCTGGTTGAAGAACATCCGGATCGTAATGGTTTTGTG
ATTCGTTATAATGAACCGGCAGTGGATGGTGAAAAATTCCTGAATAAACACGCCAT
CGAGTACCATAACATTAATCCGGATCAGTTGCAGAATTTCAGCAGATTTTATCAACA
TCCTGTATGA

pvcB

ATGGGCAGCAGCCATCATCATCATCATCACAGCAGCGGCCTGGTGCCGCGCGGC
AGCCATATGGCTAGCATGACTGGTGGACAGCAAATGGGTCGCGGATCCGAATTCA
TGAACGCATATCTGAGCGATCAGCCGGTTCGTCTGAGTCCGCTGCGTGATGAACA
GGGTAATCAGCCTCGTTTTGGTCTGCTGCTGGAACCGGGTCGTCCGGGTATGCAT
GTTGGTGAACTGCCTGCACAGTGGCTGAAAGGTCTGGCACGTAGCCATCATTTACT
GCTGCTGCGTGGTTTTGCAGCATTTGCAGATGCAGAAAGCCTGACCCGTTATTGTC
ATGATTTTGGTGAAGTTATGCTGTGGCCGTTTGGTGCAGTTCTGGAACTGGTTGAA
CAAGAAGGTGCAGAAGATCATATTTTCGCCAATAACTATGTTCCGCTGCATTGGGA
TGGTATGTATCTGGAAACCGTTCCGGAATTTCAGGTTTTTCATTGTGTTGATGCACC
GGGTGATAGTGATGGTGGTCGTACCACCTTTAGCAGCACACCGGCAGCACTGCAG
CTGGCAGATAGCAGCGAACTGGAACTGTGGCGTCGTGCAAGCGGTCGTTATCAGC
GTAGCGCAGCACATTATAGCAGCCGTAGTGCAGCACCGATTGTTGAACGTCATCC
GCGTCGTGAATTTCCGATTCTGCGTTTTTGTGAACCGGCCTGGTTGAAGGTGGATG
CAAGCTTTATCAATCCGGAGCGAAATTTCACTATGATGGGTAATTGGCACCGGGAA
CAGCGGTGGCGGAACTGCTGGGCAAGGCTGCGTCGTTGTTCTGTTATCATCCCGC
AAGGCCATTATGGCACATCGTTTGGGCGTAAGTGA

pisnB-Ah

ATGGGCAGCAGCCATCATCATCATCATCACAGCAGCGGCCTGGTGCCGCGCGGC
AGCcatATGACCGCGTCTATCGTTACCCTGGGTGGTGAACGTTGCCGTATCCTGCC
GCAGGCTCCGTTCGGTCTGCTGATCGAACCGACCCGTAACGGTCAGCCGGTTCAG
AGCCTGGCGATCGAAGCTCTGCGTGAACTGGCGCGCCGTCACGGCGTTCTGATC
CTGCGTGGTTTCGAATCTGGTTTCACTGACGCTGAACGTCTGACCCGTTACGGTGA
AGAATGGGGCGAAATCATGATGTGGCCGTTCGGCGCTGTTCTGGATGTGAAAGAA
CACGAAAACGCGACCGACCACATCTTCGACTCTAGCTACGTGCCGCTGCACTGGG
ACGGCATGTACAAACCGACCCTGCCAGAGTTCCAGCTGTTCCACTGTGTTCACGC
ACCGGCGGCTGACGAAGGTGGTCGTACCACCTTCATCAACACCCGTCAGCTGCTG

TCTGAACTGGACGGTGAACGCCTGGCGCGCTGGGAACGCGTTCACATCACCTACC
GTATCAAACAGGTGGTTCACTACGGTGGTCAGGTTAGCTCTCCGCTGCTGGTGCC
GCACCCGGTTTCCGGCGAAACTGTTCTGCGTTACAACGAACCGCCGCGTGAAGGT
GTTCGTTTCCTGAACCAGCACGCGCTGGACATCGAAGGCGTGGCGCCGGCTGAA
CAGGCAGCGTTCCTCCAGGACCTGCACCAGCGTCTGTACGACCCGCGTTACTTCT
ACGCGCACCAGTGGCAGGGTGGTGATGTTGTTATCGCTGATAACCTGGGTCTGCT
GCACGGTCGTGAAGGTTTCACCGCGCGTTCCGCGCGTCACATCCAGCGTGTTCAC
ATCCAGGCTTCTCCGGTTTGCCTGAACCCGGCTCTGGCGCCGCAGGGTGCTGCGT
AA

The plasmid encoding *pisnB*, *pvcB* or *pisnB-Ah* gene was transformed into *E. coli* BL21 (DE3) cells (New England Biolabs, MA). A single colony was picked and incubated with 100 mL Luria-Bertani (LB) and 100 µL kanamycin at 37 °C for ~ 16 hours. The cells were used as starting culture for large-scale expression with a volumetric ratio of 1:80 of starting culture to growth media. After inoculation, the cells were growing at 37 °C. Upon optical density at 600 nm (OD_{600}) reached of ~ 0.6, IPTG with final concentration of 1.0 mM was added to the culture. The cells were growing at 18 °C for 15 hours before harvesting by centrifugation at 8°C. To obtain the protein, the cells were suspended in an ice-chilled buffer (100 mM Tris, pH 7.5), and lysed by sonication. The resulting lysate was subjected to centrifugation for 30 minutes at 22,000 rpm at 4 °C, and the supernatant was loaded onto a Ni-NTA agarose column. The column was washed with 6 volumes of buffer containing 10 mM imidazole (100 mM Tris, pH 7.5). Subsequently, the desired protein was eluted using buffer containing 250 mM imidazole (100 mM Tris, pH 7.5). Fractions containing desired protein were observed by sulfate–polyacrylamide gel electrophoresis (SDS-PAGE), and concentrated to a volume of approximately 3 mL using Pull® 10K centrifugal filter. The protein solution was then dialyzed against 2 L of buffer with 5 mM EDTA and 100 mM Tris (pH 7.5), and then twice against 2 L of 100 mM Tris buffer (pH 7.5). Protein concentration was determined by UV absorption at 280 nm using a calculated molar absorptivity of 43890, 41160 and 41035 $M^{-1}cm^{-1}$ for PIsnB, PvcB and PIsnB-Ah, respectively (http://ca.expasy.org). The purities of proteins were shown by SDS–PAGE, and the gel is visualized using Coomassie-stain (Supplementary Fig. 14).

1.4 *In vitro* assays of PvcB, PIsnB and PIsnB-Ah catalyzed reactions

Liquid chromatography with detection by mass spectrometry (LC-MS) or UV-Vis was carried out on an Agilent Technologies (Santa Clara, CA) 1200 system coupled to an Agilent Technologies 6120 quadrupole mass spectrometer or an Agilent Technologies 1260 DAD detector. The associated Agilent MassHunter and OpenLAB software package were used for data collection and analysis. Detection was performed under electrospray ionization in negative mode (ESI^-). The drying gas temperature was 350 °C with a nebulizer pressure of 35 psi and flow rate of 12 L/min. The capillary voltage is set to 3000 V. Reaction mixtures were separated on a Merck SeQuant® ZIC®-cHILIC column (150 x 2.1 mm, 3.0 µm particle size). A gradient elution using solvent A (20 mM ammonium acetate aqueous solution) and solvent B (acetonitrile) with a flow rate of 0.35 mL/min was

applied. Starting with an isocratic system of 10% solvent A and 90% solvent B, followed by a gradient of 90-60% solvent B from 4 to 8 minutes. The system was then kept isocratic with 60% solvent B from 8 to 13 minutes, and then a gradient from 60-90% solvent B was applied from 13 to 17 minutes. The column was allowed to re-equilibrate for 9 min under initial conditions before subsequent sample injections. For the HPLC-condition associated with Supplementary Fig. 8A and 13B, assay mixtures were separated on an Agilent ZORBAX Extended-C18 column (4.6 ×50 mm, 1.8 μm particle size). An isocratic elution using solvent A (20 mM ammonium acetate aqueous solution) and solvent B (acetonitrile) with a flow rate of 0.4 mL/min was applied.

Reactions associated with Fig. 2 were performed as described below. Reaction mixtures including enzyme, Fe(II), substrate, 2OG, and ascorbate with the final concentration of 0.12 mM enzyme, 0.1 mM Fe(II), 3 mM 2OG, 0.5 mM substrate, and 2 mM ascorbate with final volume of 200 μL in 50 mM Tris (pH 7.68) were prepared. Reactions were carried out at 4 °C. Reaction samples were quenched using 200 μL of acetonitrile at 10 mins (Fig. 2A, 2B and 2C). Prior to LC-MS analysis, all samples were centrifuged at 14,500 rpm for 30 minutes to remove the protein.

Reactions associated with Supplementary Fig. 6A and 6B were performed analogously. Reaction mixtures containing 0.096 mM PlsnB mutant, 0.08 mM Fe(II), 1.2 mM 2OG, 0.8 mM substrate, and 0.67 mM ascorbate with final volume of 200 μL in 50 mM Tris (pH 7.68) were prepared. For the PlsnB-Ah mutant studies (Supplementary Fig. 6C), reaction mixtures containing PlsnB-Ah mutant, 0.1 mM Fe(II), 3 mM 2OG, 0.5 mM substrate, and 2 mM ascorbate with final volume of 200 μL in 50 mM Tris (pH 7.68) were prepared.

Reactions associated with Supplementary Fig. 8A were performed as described below. Reaction mixtures containing enzyme, Fe(II), substrate or analog (**4**, **5**, **6** or **7**), 2OG, and ascorbate with the final concentration of 0.12 mM protein, 0.1 mM Fe(II), 4 mM 2OG, 2 mM substrate, and 2 mM ascorbate with final volume of 200 μL in 50 mM Tris (pH 7.68) were prepared. The reactions were kept on ice for 1.5 hours. The concentration of the substrate was determined by ^{1}H-NMR, wherein 25 mM of 1,4-dioxane is used as the internal standard. The level of substrate consumption was determined by measuring the change of the peak area, in which the control experiments were carried out in the absence of 2OG.

Reactions associated with Supplementary Fig. 8B were performed as described below. Reaction mixtures enzyme, Fe(II), substrate analogs (**4**, **5** or **6**), 2OG, and ascorbate with the final concentration of 0.12 mM protein, 0.1 mM Fe(II), 0.75 mM 2OG, 0.5 mM substrate analogs, and 0.5 mM ascorbate with final volume of 200 μL in 50 mM Tris (pH 7.68) were prepared. The reactions were kept on ice for 1.5 hours.

Reactions associated with Supplementary Fig.10 were performed in a similar manner. Reaction mixtures containing 0.24 mM PlsnB, 0.20 mM Fe(II), 3 mM 2OG, 2 mM substrate, and 1 mM ascorbate with final volume of 200 μL in 50 mM Tris (pH 7.56) were prepared.

Reactions associated with Supplementary Fig.13A were performed as described below. Reaction mixtures containing enzyme, Fe(II), **9** or **10** and ascorbate with the final concentration of 0.12 mM protein, 0.1 mM Fe(II), 2.0 mM **9** or **10**, and 0.5 mM ascorbate with final volume of 200 µL in 50 mM Tris (pH 7.5) were prepared. The reactions were kept on ice for 16 hours before quenched with 200 µL of acetonitrile. The samples were analyzed by UV-Vis detected at 266 nm.

Reactions associated with Supplementary Fig. 13B were performed following the conditions of Supplementary Fig. 8A. In addition to the substrate (**4**), an equal molar of the analog (**9** or **10**) was also added the reaction mixture. The reactions were initiated by addition of 2OG. The control experiments were carried out in the absence of 2OG.

1.5 Using ^{13}C-NMR spectroscopy to follow enzymatic reactions

Reaction mixtures containing protein (PlsnB or PvcB), Fe(II), [2-^{13}C]-**6**, 2OG, and ascorbate with the final concentration of 0.48 mM protein (PlsnB or PvcB), 0.4 mM Fe(II), 12.0 mM 2OG, 2.0 mM [2-^{13}C]-**6**, and 8.0 mM ascorbate with final volume of 600 µL in 50 mM Tris (pH 7.68) were prepared. The reaction mixtures were shaking with a speed of 220 rpm for 17 hours at 18°C. Prior to NMR measurement, 30 µL of DMSO-d_6 was added to the reaction followed by centrifugation at 14,500 rpm for 30 minutes. The supernatant was then transferred to the NMR tubes. The ^{13}C-NMR spectra were recorded using Bruker NEO 700 MHz. The NMR spectra are shown in Fig. 5.

1.6 Differential scanning fluorimetry assay to measure protein stability

Purified PlsnB in the concentration of 5 to 20 µM was pre-incubated with 2.5 mM $MnCl_2$ or 2 mM compound **4** on ice for 30 minutes in 96-well low-profile PCR plates (ABgene, Thermo Scientific). 10X SYPRO® Orange (Molecular Probes) was added into each well and mixed prior to measurement in an RT-PCR machine (LightCycler 480, Roche). The protein melting experiments were carried out with a continuous temperature acquisition mode using 10 acquisitions per 1ºC in each cycle from 20°C to 95°C. The melting curves of the PlsnB were monophasic and T_m values were derived using Boltzmann equation[11].

1.7 Crystallization and X-ray crystallography

Protein crystallization

In order to identify crystallization conditions for PlsnB, 12 mg/mL of purified PlsnB protein sample was incubated overnight with 2.5 mM Mn^{2+} ion and 2 mM **4** before the screening in sparse matrix with a Phoenix crystallization robotic system (Art Robbins Instruments). After two-weeks incubation of screening trays at 4°C, rod shaped crystals appeared in a condition that contains 0.1 M MES (pH 6.5), 0.2 M ammonium sulfate, and 30% PEG 5000MME. This crystallization condition was further optimized by manually setting sitting-drop vapor diffusion experiments with varying pH and precipitant concentration, resulting diffraction-quality crystals.

Data collection and structure determination

Individual crystals were flash-frozen directly in liquid nitrogen after brief incubation with a reservoir solution supplemented with 30% (v/v) glycerol. X-ray diffraction data were collected at 23-ID-B beamline in Advance Photon Source (Lemont, IL). By using HKL2000[12], X-ray diffraction pattern was processed to 1.98 Å resolution for PIsnB•Mn complexed with **4**. In Phenix software[13], phases were obtained by molecular replacement using a previously obtained PvcB structure as the initial search model (PDB code 4YLM). The molecular replacement solution for PIsnB structure were iteratively built using Coot[14] and Phenix refinement package. The omit map was first calculated with only the protein information, revealing a strong positive density close to the metal binding center. Different chemical compounds used during the expression and purification was manually built inside the density with Coot [14] to compare the consistency of the density with the chemical molecule. The 2FoFc and FoFc maps were generated with the most likely candidates, as shown in Supplementary Fig. 4C-E. The quality of the finalized crystal structure was evaluated by MolProbity and ProCheck. The final statistics for data collection and structural determination are shown in Table S1.

<u>Substrate docking</u>

The binding models for PIsnB with different ligands (**5**-**8**) were obtained by using the complex structure of PIsnB and **4** as an initial reference. The binding mode was then optimized with Maestro (Schrodinger, LLC)[15] which features a minimization routine based on OPLS_2005 Forcefield[16-17]. A substrate binding model of PvcB was conducted in a similar method using apo PvcB structure (PDB code: 4YLM). The initial position of the substrate were obtained by the superimposition of apo PvcB and PIsnB with substrate complex. The model is then subject to energy minimization in Maestro.

<u>1.8 Sequence alignment and analysis</u>

Based on the structural scaffolds of natural products isolated in rhabduscin[3], byelyankacin[3-5], xanthocillin[6-7], paerucumarin[8-9], and **12**[10] (the structure are shown in Supplementary Fig.11A), the Fe/2OG enzymes responsible for vinyl isonitrile or isocyanoacrylate formation are categorized into PIsnB- or PvcB-type enzymes, respectively. A Basic Local Alignment Search Tool (BLAST) search using amino acid sequence of PIsnB from *Photorhabdus luminescens* was used as query to search for corresponding enzymes involved in each natural product biosynthesis. A sequence alignment analysis of these Fe/2OG enzymes is shown in Supplementary Fig. 11B.

2. Supplementary Figures

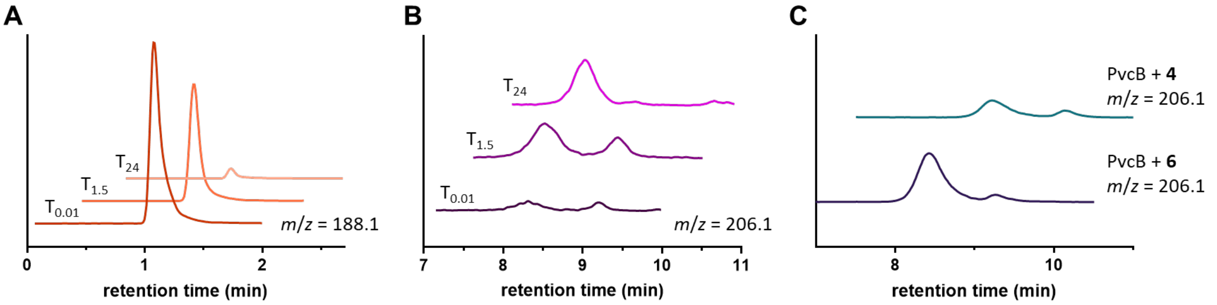

Supplementary Figure 1. (A) and (B) PvcB catalyzed reactions using **4** as a substrate were quenched at different time points (0.01, 1.5 or 24 hours). As the incubation time increased, isocyanoacrylate **2** (*m*/*z* = 188.1, panel A) was hydrolyzed to the corresponding *N*-formylacrylate (*m*/*z* = 206.1, panel B). (C) Alignment of PvcB-catalyzed reactions using **4** or **6** as a substrate. The resulting hydrolyzed product from isocyanoacrylate **2** has the same retention time as of the reaction product formed using **6** as a substrate.

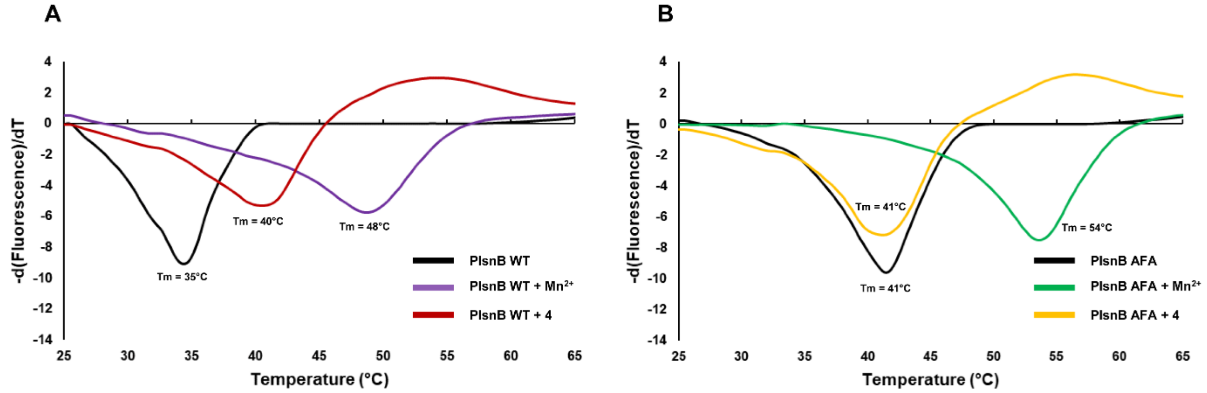

Supplementary Figure 2. The stabilization effect of ligand/ion binding on PlsnB. (A) Representative DSF analysis of the stabilizing effect of ligand and metal on PlsnB. (B) Representative DSF analysis of the stabilizing effect of ligand and metal on PlsnB mutant (M105A_Y106F_K107A). Each sample was setup with technical triplets. Due to batch to batch differences, the T_m for each sample differs after freeze/thaw cycle but the ΔT_m before and after ligand incorporation is always consistent.

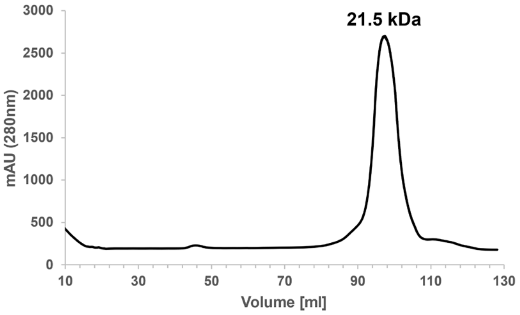

Supplementary Figure 3. Size-exclusion chromatography profile of purified recombinant PlsnB.

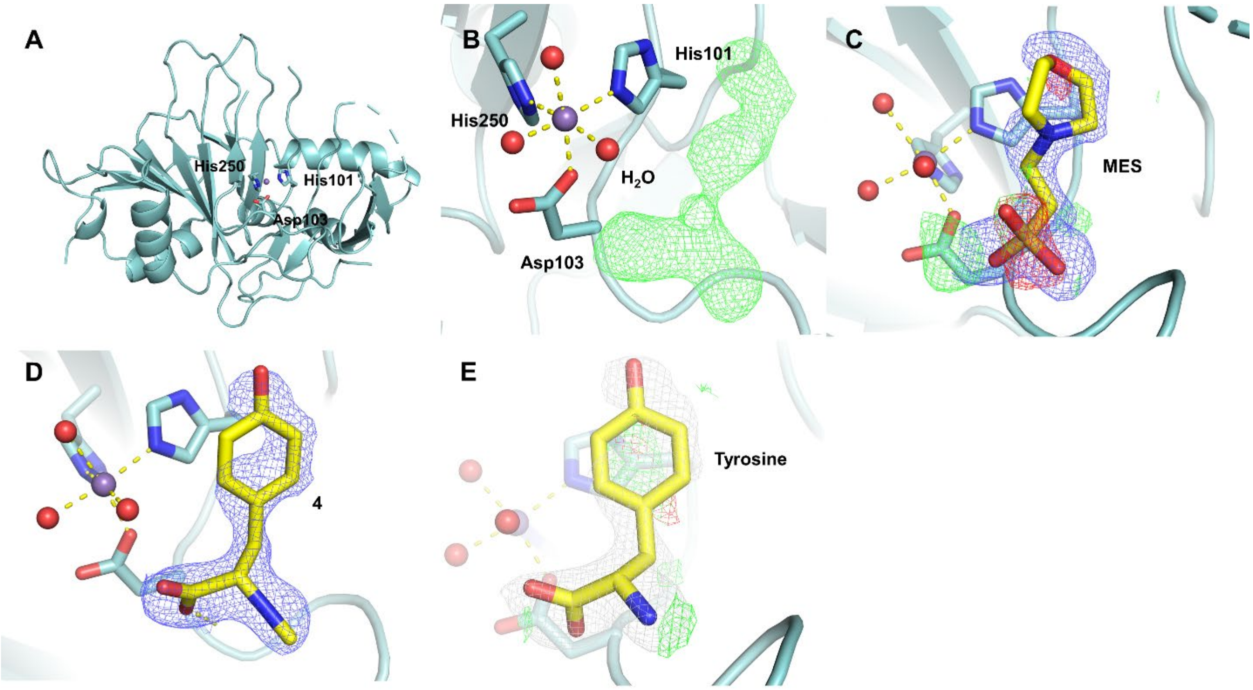

Supplementary Figure 4. The complex structure of PlsnB•Mn and compound **4**. (A) Overall fold of PlsnB enzyme shown in ribbon diagram. The triad residues coordinating metal ion are shown in sticks. The disordered region is shown as dashed lines. (B) The omit map at the active site of PlsnB co-crystallizing with compound **4**. The green mesh shows the positive density of FoFc omit map contoured to 3 σ. (C) 2FoFc and FoFc maps at the active site of PlsnB with 2-(*N*-morpholino)ethanesulfonic acid built into the density. The green mesh shows the positive density of FoFc omit map contoured to 3 σ, red mesh for negative density contoured to -3 σ. The blue mesh exhibits 2FoFc map contoured to 1 σ. (D) 2FoFc map at the active site of PlsnB with the substrate **4** built into the density. (E) 2FoFc and FoFc maps at the active site of PlsnB with L-tyrosine built into the density. The 2FoFc map is shown in grey mesh at 1 σ. The positive density is colored green with a contour of 3 σ.

```
WP_011147037.1          YRINKVTHYGG-----EVHSPLVEEHPDRNGFVIRYNE  189
WP_010845413.1          YRTKKVTHYGG-----EVVSPLVCLHPKGNKWVIRYNE  190
WP_033651693.1          YRIKQVVHYGG-----EVCSPLVVQHPNGRGLIMRYNE  190
WP_080516319.1          YRIKAVVHYGG-----EVTSPLVIPHPNGTGEIMRYNE  185
CBF87187.1              FWDAKVYNLPL-----------VIKHPDTGLPSLRWHQ  585
MT229078 (KZN89140.1)   FWDAKLKNLRL-----------IVRHPVSGLPCLRWHQ  619
WP_001146424.1          YQRKMEFYHSK------TVSPIVMQHPYRDYQVIRYNE  183
AAC21672.1              YQRSAAHYSSR------SAAPIVERHPRREFPILRFCE  197
WP_004188574.1          YRRTVELYSNT------VEAPIVERHPRREFPILRFCE  197
WP_004157574.1          YQRSVELYSNT------VEAPIIGIHPLREFPVIRFCE  197
WP_017765143.1          YRIKQVVHYGG-----QVSSPLLVPHPVSGETVLRYNE  195
```

Supplementary Figure 5. Sequence alignment of several Fe/2OG enzymes that catalyze vinyl isonitrile or isocyanoacrylate formation. A ''capping loop'' (I159-V162) suggested in the PIsnB ternary complex structure is not conserved among Fe/2OG enzymes. The ''capping loop'' region is highlighted. The NCBI reference sequence numbers for PIsnB and PvcB are WP_011147037 and AAC21672, respectively.

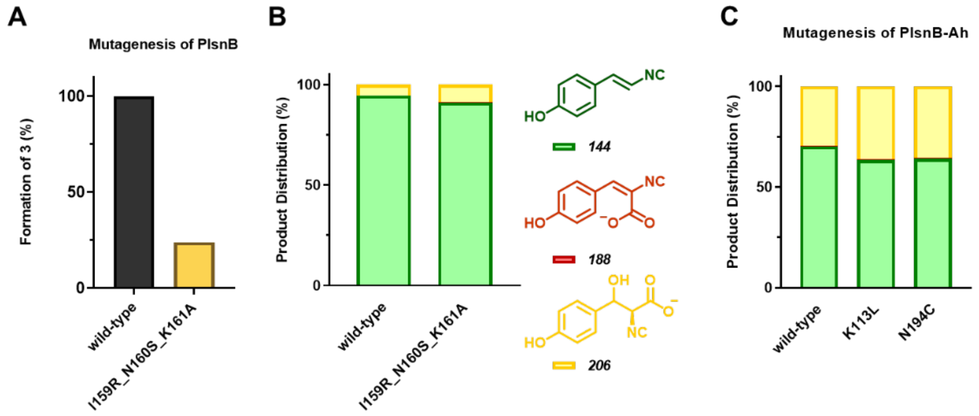

Supplementary Figure 6. Mutagenesis studies of PIsnB (A and B) and PIsnB-Ah (C). (A) and (B) PIsnB mutant was purified and characterized. Under the current experimental conditions, while this variant is less active than the wild-type, the product distribution is not changed. (C) Variants of PIsnB-Ah exhibit similar product profile as of the wild-type. Experiments were repeated two times independently with similar results.

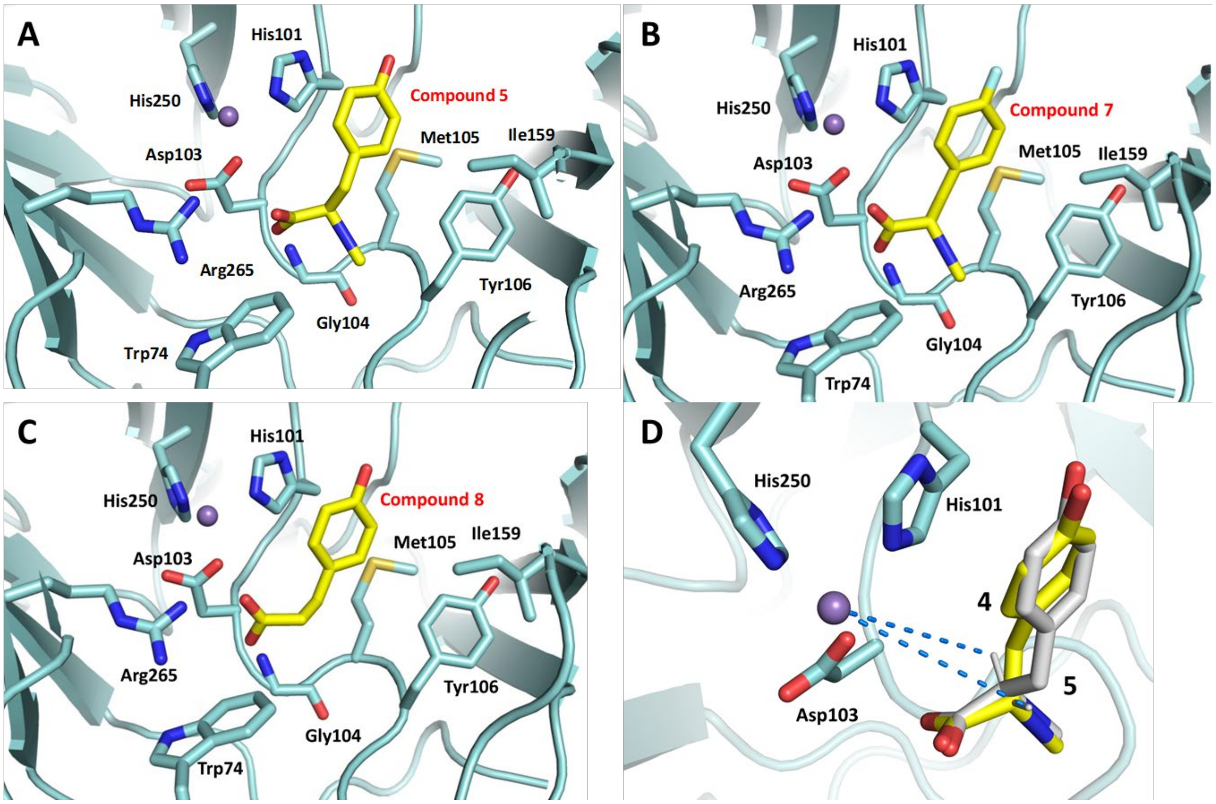

Supplementary Figure 7. (A),(B) and (C) Modelling of **5**, **7** or **8** into the active site of PlsnB using Maestro. (D) An overlay of the **4**-bound PlsnB with **5** (grey). Dashed lines (Fe-Cα-H) indicate the distance (~ 5.3 and 5.5 Å) and the positions of predicted Cα-Hs. The Cα-Hs are shown in thin sticks.

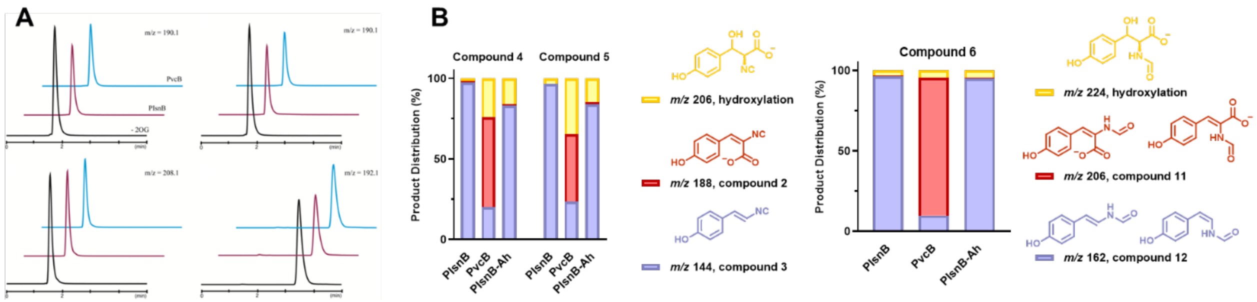

Supplementary Figure 8. (A). Substrate consumption of **4**, **5**, **6** and **7**. From the bottom to top, traces show the substrate peak at the conditions of PlsnB without 2OG (-2OG), PlsnB and PvcB. The substrates have the *m*/*z* value of 190.1, 190.1, 208.1 and 192.1 for **4**, **5**, **6** and **7**, respectively. (B) Product profiles of PlsnB, PvcB and PlsnB-Ah catalyzed reactions using **4**-**6** as substrates. Products associated with desaturation, hydroxylation and decarboxylation-desaturation are color coded with red, yellow and purple, respectively. Experiments were repeated two times independently with similar results.

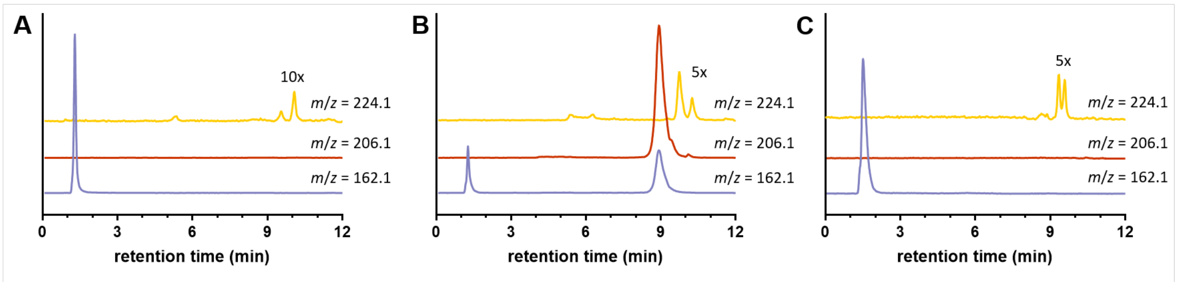

Supplementary Figure 9. LC-MS chromatograms of PlsnB, PvcB or PlsnB-Ah catalyzed reactions using **6** as the substrate. (A) In the PlsnB reaction, conversion of **6** to vinyl formyl compound **12** was identified (*m/z* 208.1 → 162.1). (B) *N*-formylacrylate **11** was detected as the major product (*m/z* 208.1 → 206.1) in the PvcB catalyzed reaction. (C) Conversion of **6** to **12** was also identified (*m/z* 208.1 → 162.1) in the PlsnB-Ah reaction. In all reactions, a minor hydroxylation product (*m/z* 224.1) was also detected. Traces for hydroxylation are magnified for clear visualization. In each panel, traces associated with the corresponding **12**, **11** and hydroxylation products are colored with purple, red and yellow, respectively. Experiments were repeated two times independently with similar results.

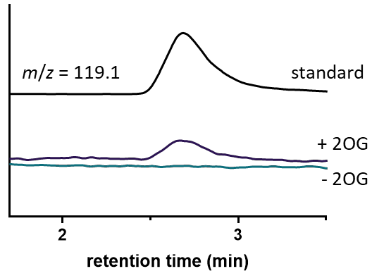

Supplementary Figure 10. LC-MS chromatograms of PlsnB-catalyzed conversion of phloretic acid (**8**) to 4-vinylphenol was identified (*m/z* 165.1 → 119.1). The peak produced in the enzymatic reaction has the same retention time and isotope distribution as the standard of 4-vinylphenol. The experiment was repeated two times independently with similar results.

A

Isolated Natural Product	Organism	NCBI Reference Sequence	Isolated Natural Product	Organism	NCBI Reference Sequence
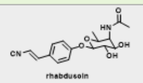	*Photorhabdus luminescens*	WP_011147037.1<br>ref [3]	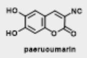	*Pseudomonas aeruginosa* PAO1	AAC21672.1<br>ref [8]
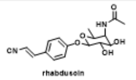	*Xenorhabdus nematophila*	WP_010845413.1<br>ref [3]	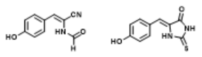	*Burkholderia mallei*	WP_004188574.1<br>ref [5]
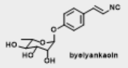	*Enterobacterales*	WP_033651693.1<br>ref [4]	Orthologue proposed to catalyzed **2** formation	*Erwinia amylovera*	WP_004157574.1<br>ref [9]
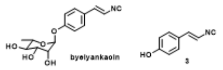	*Erwinia carotovora*	WP_080516319.1<br>ref [5]			
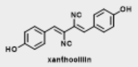	*Aspergillus nidulans* FGSC A4	CBF87187.1<br>ref [6-7]			
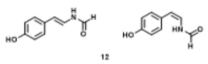	*Penicillium chrysogenum*	MT229078<br>ref [10]			
Orthologue proposed to catalyzed **3** formation	*Vibrio cholerae*	WP_001146424.1<br>ref [3]			
Orthologue used in this research	*Aeromonas hydrophila*	WP_017765143.1			

B

WP_011147037.1	101	HWDGMYK	RYN	HGREGFTSKSERHLQRI
WP_010845413.1	102	HWDGMYR	RYN	HGREAFISRSPRHLQRV
WP_033651693.1	102	HWDGMYK	RYN	HGREGFTARSARHLQRV
WP_080516319.1	97	HWDGMYK	RYN	HGREAFTAQSARHLQRV
CBF87187.1	483	HFDGMFR	RWH	HTRTAFESGCNRELWRI
MT229078 (KZN89140.1)	517	HFDGMFK	RWH	HTRTGYISDCDRELWRI
WP_001146424.1	96	HWDGMYR	RYN	HGREGFTSHTPRHIRRV
AAC21672.1	110	HWDGMYL	RFC	HGREAFAHRAPRHLRRV
WP_004188574.1	110	HWDGMYL	RFC	HGRERFTSRSGRHLRRV
WP_004157574.1	110	HWDGMYL	RFC	HGREQYTHHSGRHLRRV
WP_017765143.1	107	HWDGMYK	RYN	HGREGFTARSARHIQRV

Supplementary Figure 11. (A) Summary of PlsnB- and PvcB-type enzymes. Based on the structural scaffold identified in rhabduscin, byelyankacin, xanthocillin, paerucumarin, and **12**, the corresponding Fe/2OG enzymes are categorized into PlsnB- or PvcB-type enzymes. (B) Amino acid sequence alignments of enzymes shown in panel A. Two residues are identified to be associated with reaction selectivity. In PlsnB-type enzymes, a positively charged residue including lysine or arginine is conserved at the K107 position of PlsnB. In contrast, PvcB-type enzymes have a leucine at this position. Moreover, in the downstream region, PlsnB-type enzymes occupy a conserved residue with nitrogen-containing side chain, such as asparagine or histidine, at the N188 position of PlsnB. In contrast, PvcB-type enzymes have a cysteine at this position. Additionally, the conserved arginine residue (R265 in PlsnB) is colored in gray, while 2-histidine and 1-aspartate used for iron chelation are colored in blue.

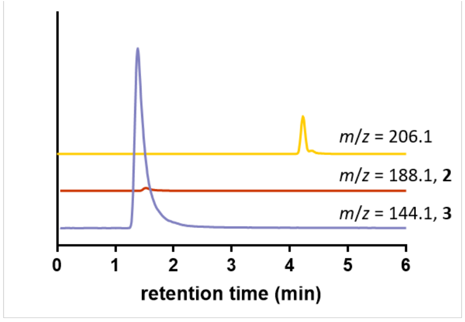

Supplementary Figure 12. LC-MS chromatograms of PIsnB-Ah-catalyzed reactions using **4** as a substrate. Conversion of **4** to vinyl isonitrile **3** along with hydroxylated compound were identified (*m/z* 190.1 → 144.1 (**3**) and 206.1). In each panel, traces associated with the corresponding vinyl isonitrile, isocyanoacrylate and hydroxylation products are colored with purple, red and yellow, respectively. Experiments were repeated two times independently with similar results.

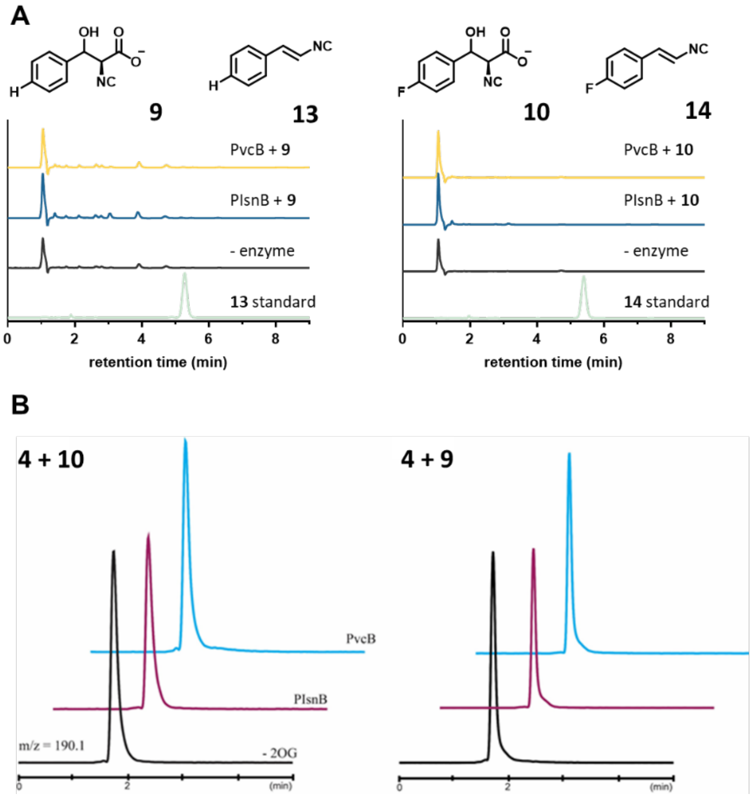

Supplementary Figure 13. (A) UV-Vis-LC chromatograms of PIsnB- and PvcB-catalyzed reactions using proposed hydroxylated intermediate (**9** or **10**) as the substrate. Under the conditions of enzyme to substrate ratio of 1:20, no obvious new peaks can be detected after 16 hours incubation. Vinyl isonitrile product standard (**13** and **14**) were also prepared and are shown in the bottom trace of each panel (λ_{266}). (B) Substrate consumption of **4** in the competition experiments using **9** or **10** (**4**+**9** or **4**+**10).** The substrate (**4**) has the *m*/*z* value of 190.1. Experiments were repeated two times independently with similar results.

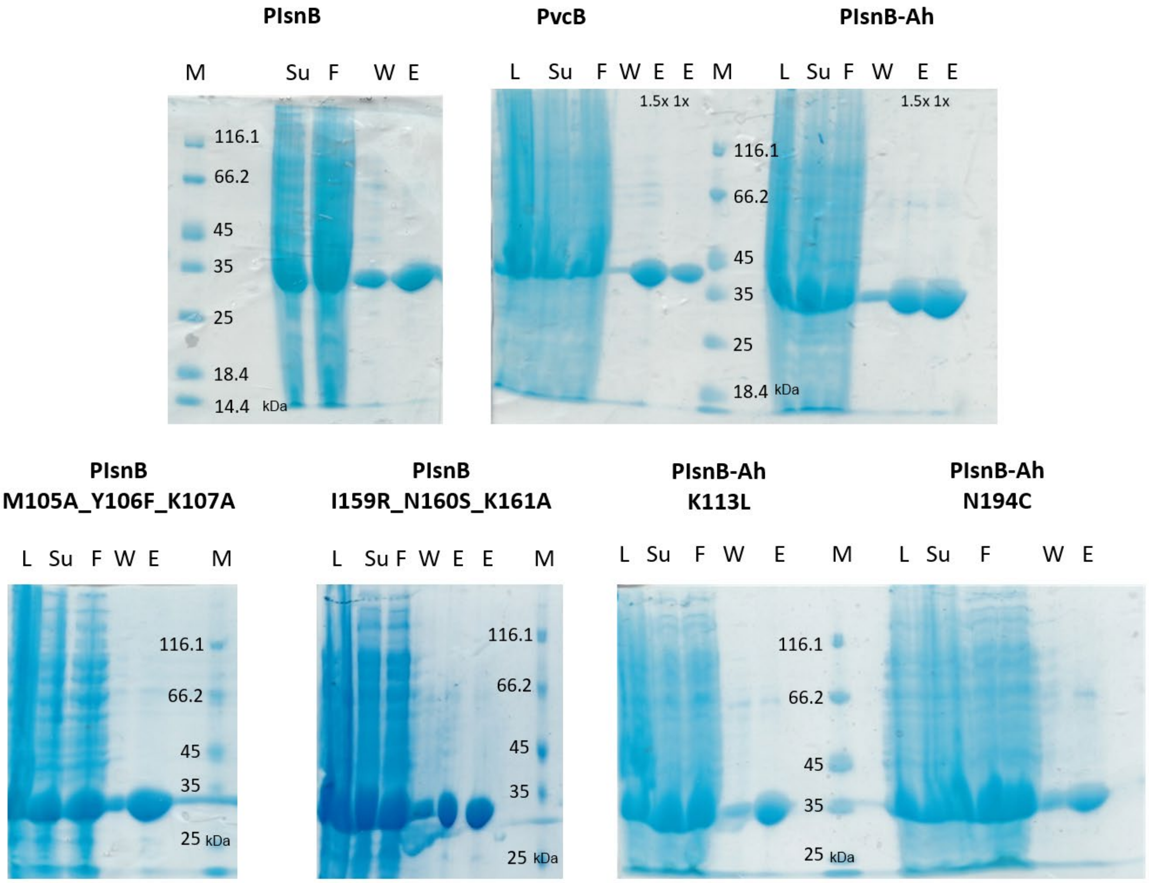

Supplementary Figure 14. Coomassie-stained SDS-PAGE (L: lysate, Su: supernatant after centrifuge, F: flow-through, W: wash, E: elution and M: marker). Units of molecular weight markers are kilodalton (kDa). Protein purification were repeated two times independently with similar results.

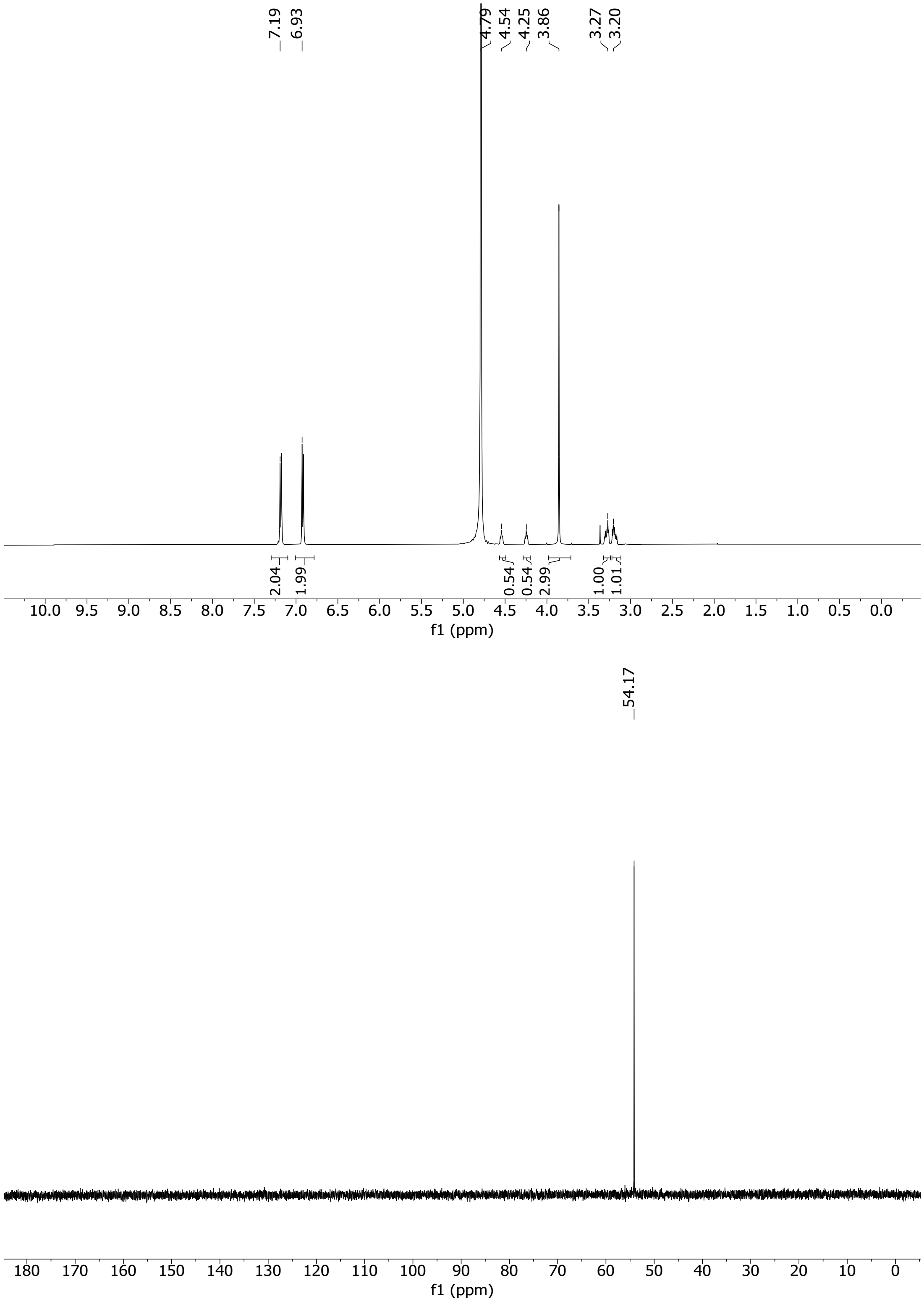

Supplementary Figure 15. ^{1}H and ^{13}C NMR of compound **15**.

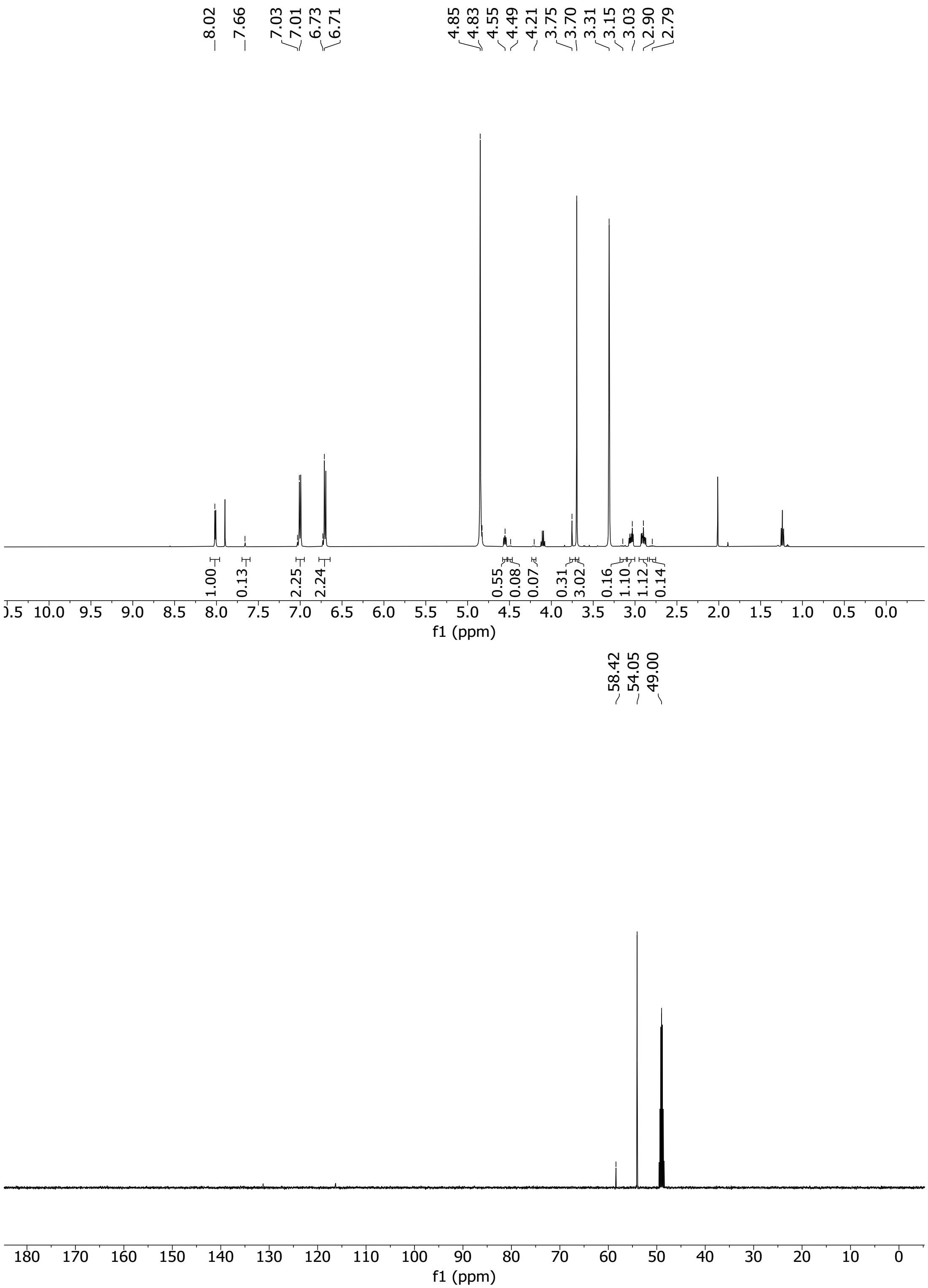

Supplementary Figure 16. ^{1}H and ^{13}C NMR of compound **16**.

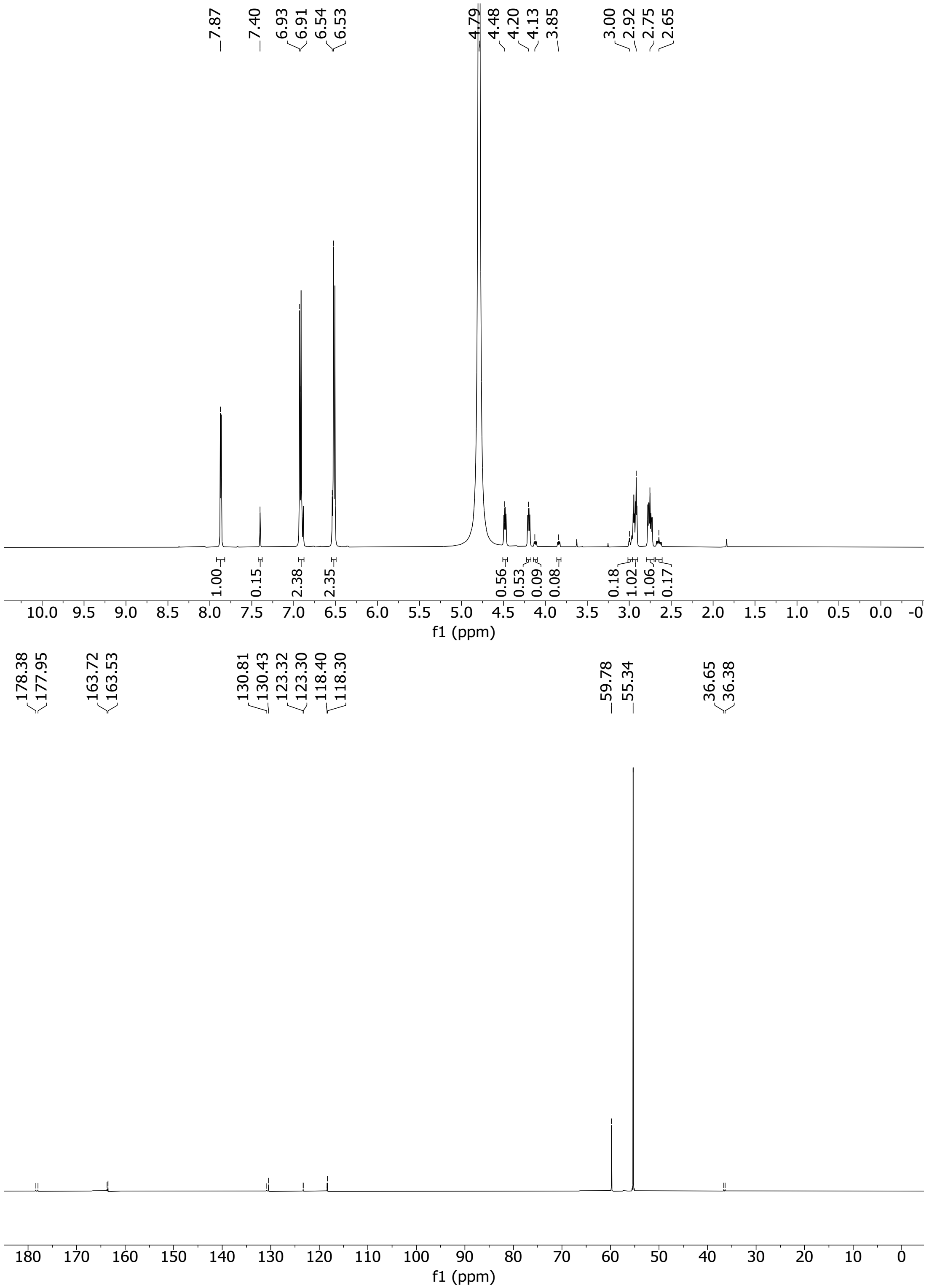

Supplementary Figure 17. ^{1}H and ^{13}C NMR of compound [2-^{13}**C**]-**6**.

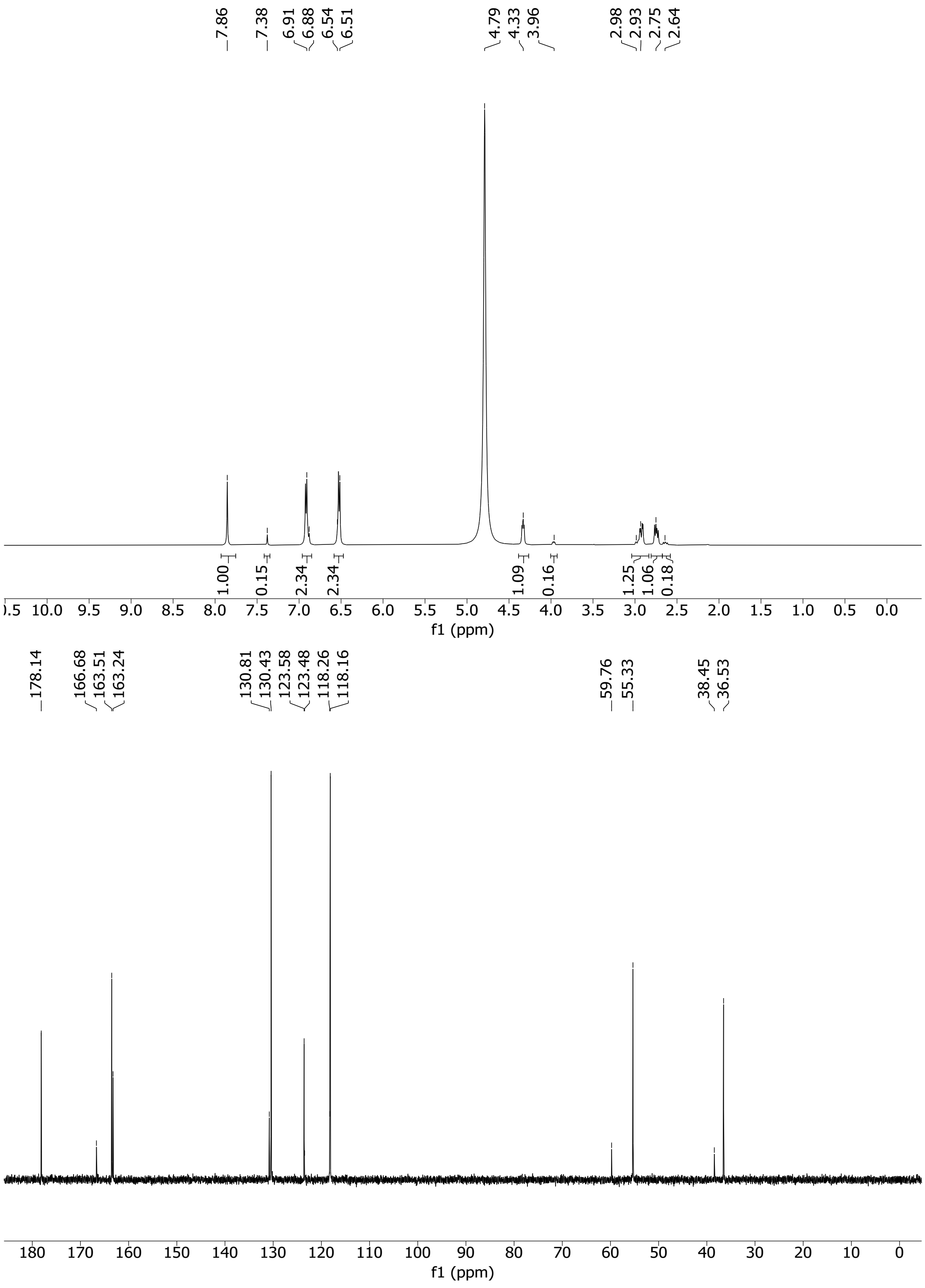

Supplementary Figure 18. ^{1}H and ^{13}C NMR of compound **6**.

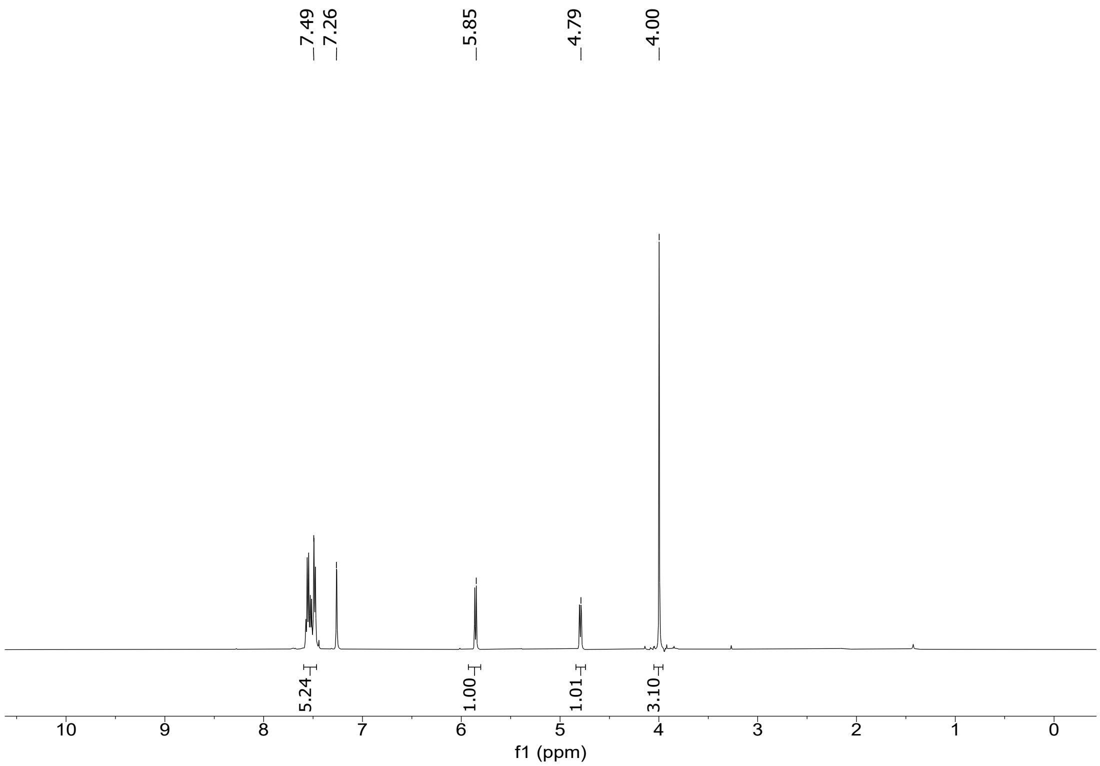

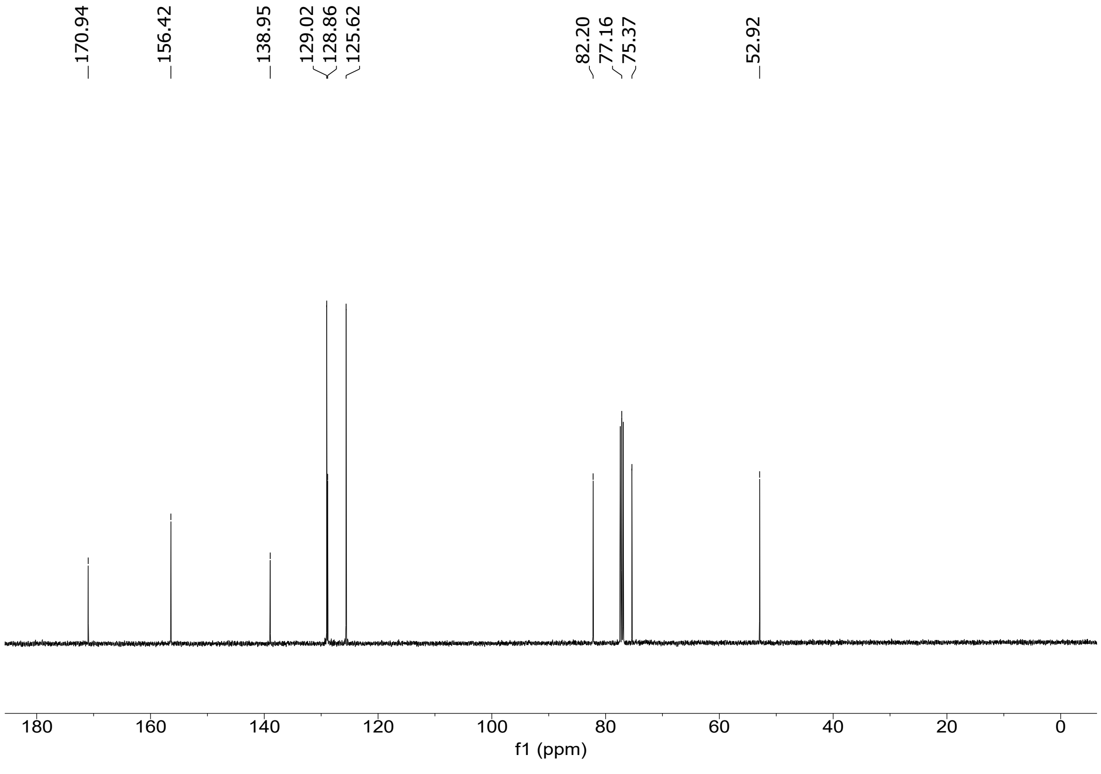

Supplementary Figure 19. ^{1}H and ^{13}C NMR of compound **17**.

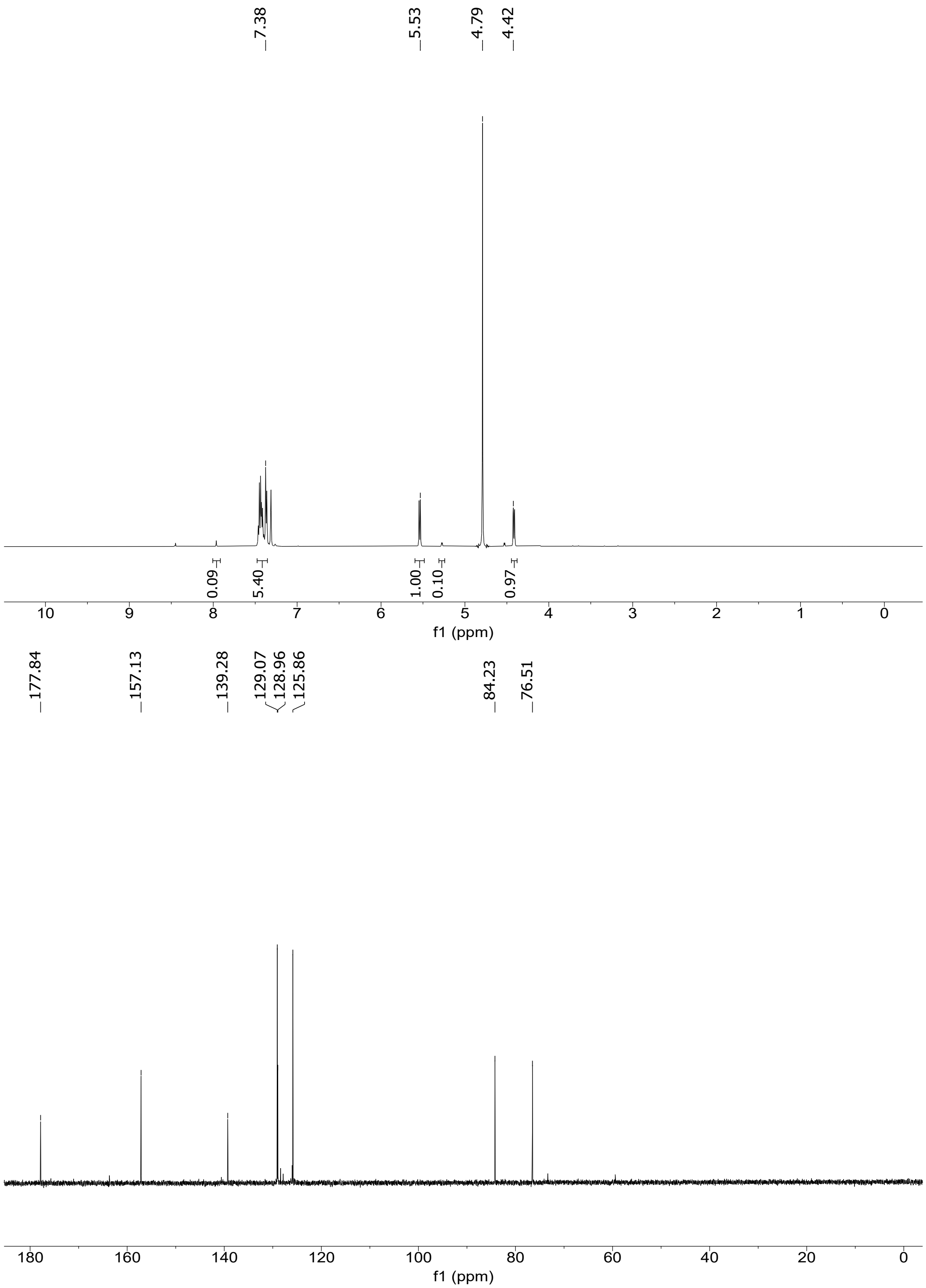

Supplementary Figure 20. ^{1}H and ^{13}C NMR of compound **9**.

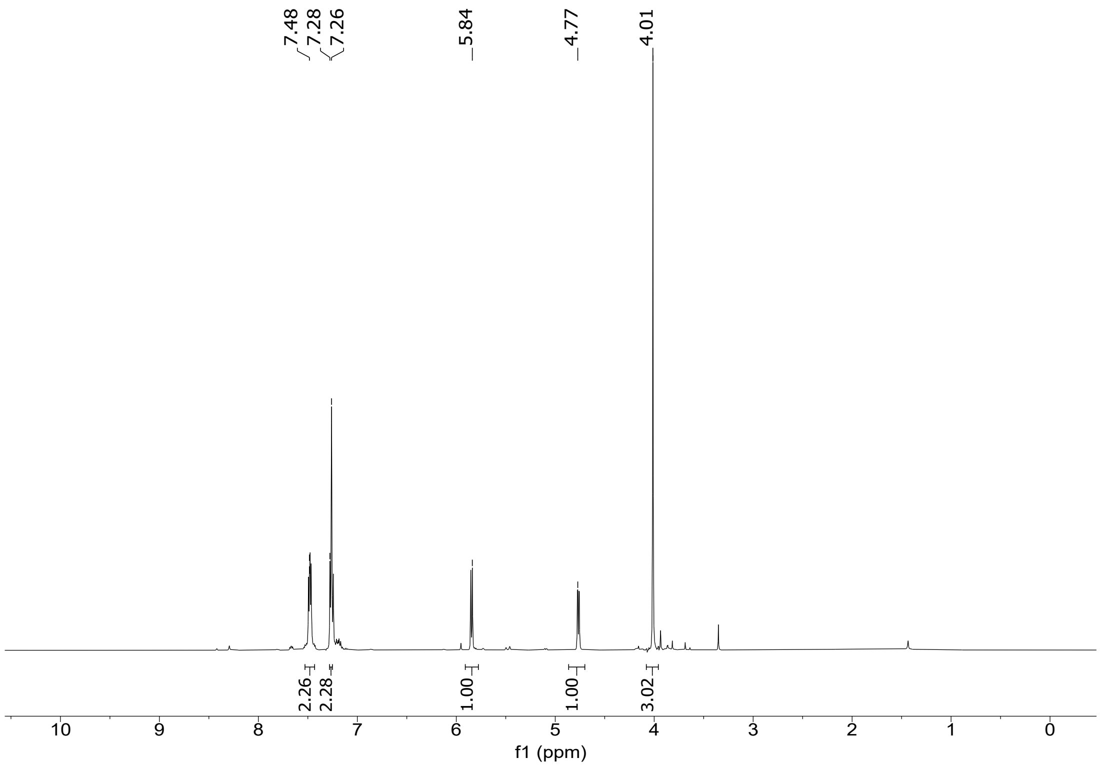

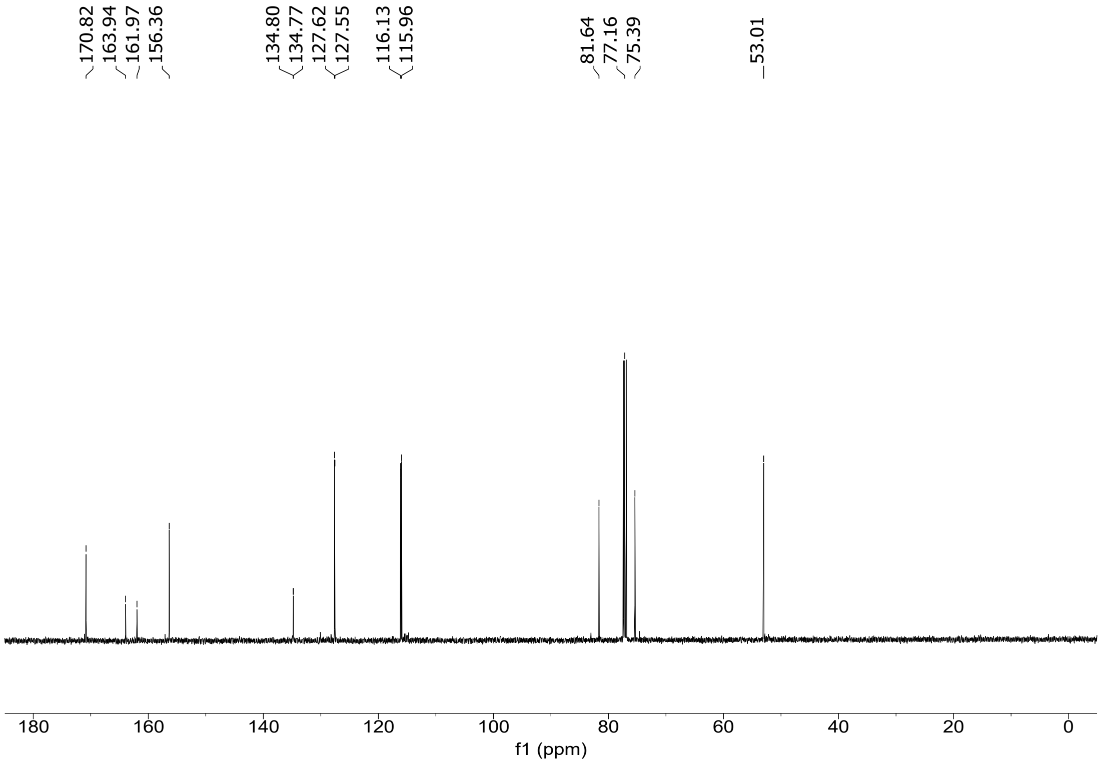

Supplementary Figure 21. ^{1}H and ^{13}C NMR of compound **18**.

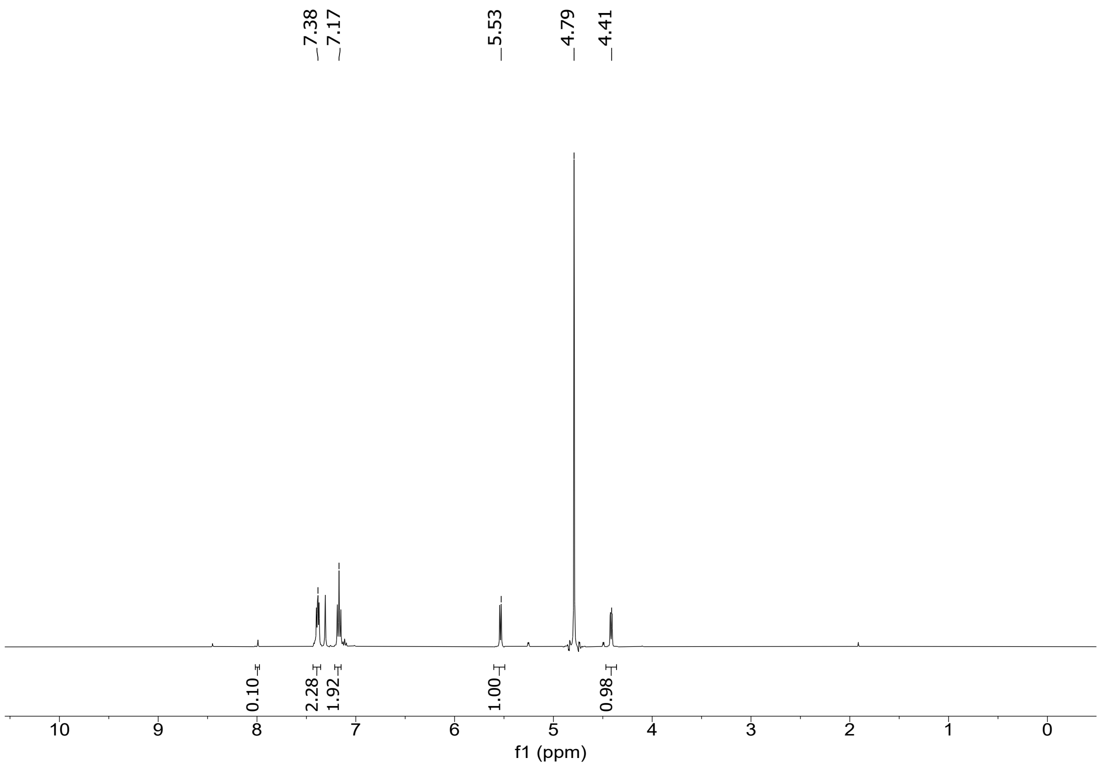

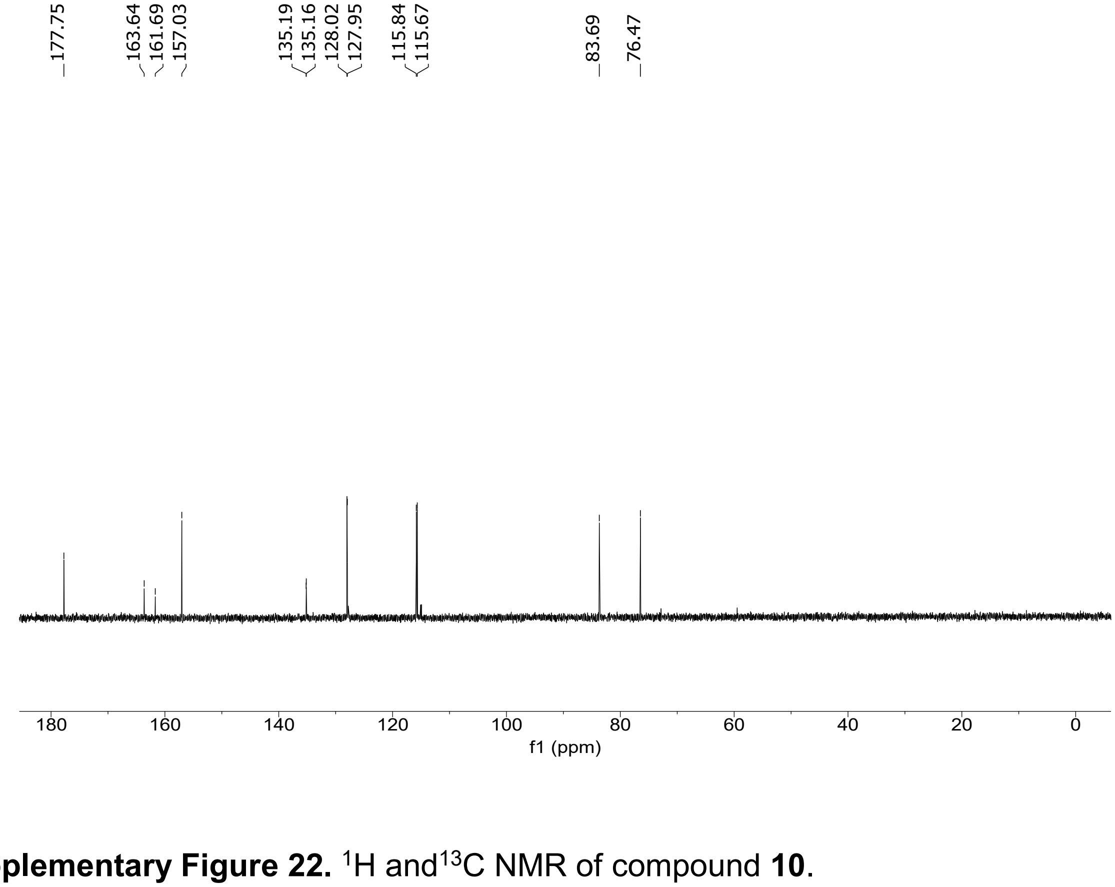

Supplementary Figure 22. ^{1}H and ^{13}C NMR of compound **10**.

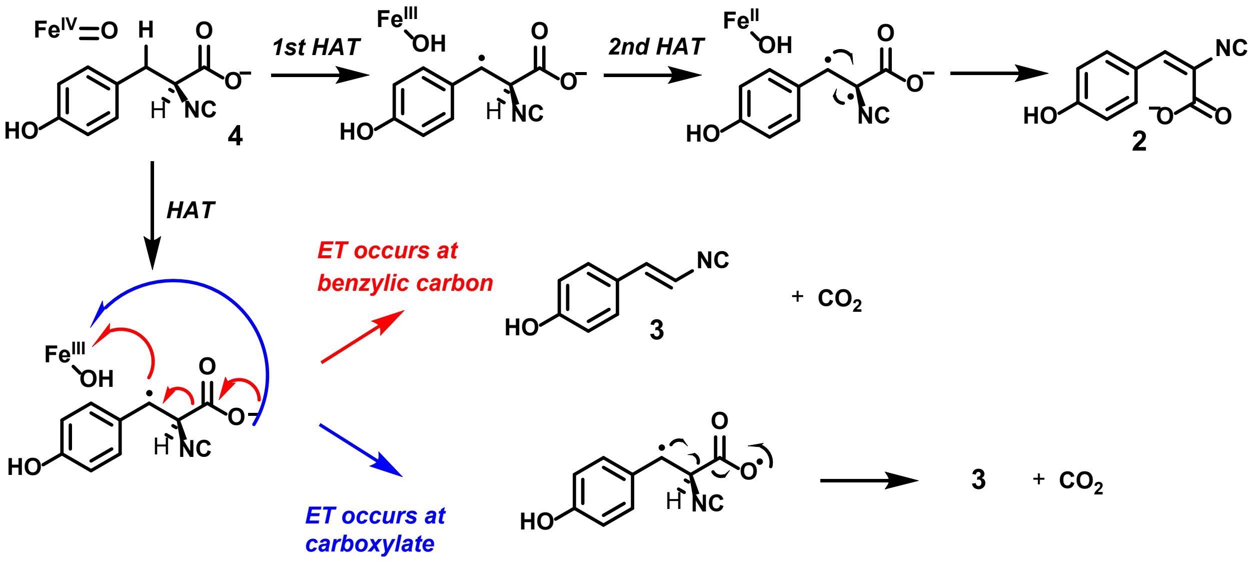

Supplementary Figure 23. Other possible mechanisms can account for PlsnB- and PvcB- catalyzed desaturations. In addition to the pathways that deploy a hydroxylated intermediate or a carbocation species (as shown in Scheme 1), pathways include two hydrogen atom abstraction (HAT) processes or electron transfer (ET) promoted C-C bond cleavage can be envisioned for the PvcB and PlsnB catalyzed reactions, respectively.

3. Supplementary Table

Supplementary Table 1. Statistics for X-ray diffraction data collection and structural determination.

**Data collection**	PlsnB•Mn•**4**
Space group	$P2_12_12_1$
Cell dimensions	
a, b, c (Å)	49.87, 58.38, 102.59
α, β, γ (°)	90.00, 90.00, 90.00
Resolution (Å)	50.00 - 1.98 (2.01 - 1.98)
R_{sym}/ R_{pim}	0.114(0.713)/0.048(0.345)
CC ½ ^{Y}	0.907 (0.702)
I / σ	13.6 (1.7)
Completeness (%)	99.4 (99.5)
Redundancy	6.3 (5.0)
**Refinement**	
Resolution (Å)	44.850 - 1.971 (2.042 - 1.971)
No. reflections	21569 (2006)
R_{work}	0.1817 (0.2468)
$R_{free}^{\pm}$	0.2124 (0.2943)
**No. atoms**	2298
Protein	2107
Ligand/ion	30
Water	161
**B-factors (Å^2)**	
Protein	29.9
Ligand/ion	40.6 / 51.7
Water	38.9
**R.m.s. deviations**	
Bond lengths (Å)	0.0069
Bond angles (°)	0.82
**Ramachandran plot**	
Favored	98.80%
Allowed	1.20%
Outliers	0.00%
**Molprobity score**	1.27 / 99th percentile

*Values for the corresponding parameters in the outermost shell in parenthesis.

$^{Y}CC_{1/2}$ is the Pearson correlation coefficient for a random half of the data, the two numbers represent the lowest and highest resolution shell respectively.

$^{\pm}R_{free}$ is the R_{work} calculated for about 10% of the reflections randomly selected and omitted from refinement.

4. Supplementary References

[1] Gottlieb, H. E.; Kotlyar, V.; Nudelman, A., NMR Chemical Shifts of Common Laboratory Solvents as Trace Impurities. *J. Org. Chem.* **1997**, *62*, 7512-7515.

[2] Yu, C. P.; Tang, Y.; Cha, L.; Milikisiyants, S.; Smirnova, T. I.; Smirnov, A. I.; Guo, Y.; Chang, W. C., Elucidating the Reaction Pathway of Decarboxylation-Assisted Olefination Catalyzed by a Mononuclear Non-Heme Iron Enzyme. *J. Am. Chem. Soc.* **2018**, *140*, 15190-15193.

[3] Crawford, J. M.; Portmann, C.; Zhang, X.; Roeffaers, M. B.; Clardy, J., Small molecule perimeter defense in entomopathogenic bacteria. *Proc. Natl. Acad. Sci. U. S. A.* **2012**, *109*, 10821-10826.

[4] Takahashi, S.; Iwai, H.; Kosaka, K.; Miyazaki, T.; Osanai, Y.; Arao, N.; Tanaka, K.; Nagai, K.; Nakagawa, A., Byelyankacin: A Novel Melanogenesis Inhibitor Produced by Enterobactersp. B20. *J. Antibiot.* **2007**, *60*, 717-720.

[5] Brady, S. F.; Bauer, J. D.; Clarke-Pearson, M. F.; Daniels, R., Natural Products from isnA-Containing Biosynthetic Gene Clusters Recovered from the Genomes of Cultured and Uncultured Bacteria. *J. Am. Chem. Soc.* **2007**, *129*, 12102-12103.

[6] Lim, F. Y.; Won, T. H.; Raffa, N.; Baccile, J. A.; Wisecaver, J.; Rokas, A.; Schroeder, F. C.; Keller, N. P., Fungal Isocyanide Synthases and Xanthocillin Biosynthesis in Aspergillus fumigatus. *mBio* **2018**, *9*.

[7] Raffa, N.; Won, T. H.; Sukowaty, A.; Candor, K.; Cui, C.; Halder, S.; Dai, M.; Landero-Figueroa, J. A.; Schroeder, F. C.; Keller, N. P., Dual-purpose isocyanides produced by Aspergillus fumigatus contribute to cellular copper sufficiency and exhibit antimicrobial activity. *Proc Natl Acad Sci U S A* **2021**, *118* (8).

[8] Clarke-Pearson, M. F.; Brady, S. F., Paerucumarin, a new metabolite produced by the pvc gene cluster from Pseudomonas aeruginosa. *J. Bacteriol.* **2008**, *190*, 6927-6930.

[9] Zhu, J.; Lippa, G. M.; Gulick, A. M.; Tipton, P. A., Examining Reaction Specificity in PvcB, a Source of Diversity in Isonitrile-Containing Natural Products. *Biochemistry* **2015**, *54*, 2659-2669.

[10] Khan, I.; Zhang, H.; Liu, W.; Zhang, L.; Peng, F.; Chen, Y.; Zhang, Q.; Zhang, G.; Zhang, W.; Zhang, C., Identification and bioactivity evaluation of secondary metabolites from Antarctic-derived Penicillium chrysogenum CCTCC M 2020019. *RSC Advances* **2020**, *10* (35), 20738-20744.

[11] Niesen, F. H.; Berglund, H.; Vedadi, M., The use of differential scanning fluorimetry to detect ligand interactions that promote protein stability. *Nat. Protoc.* **2007**, *2*, 2212-2221.

[12] Otwinowski, Z.; Minor, W., Processing of X-Ray Diffraction Data Collected in Oscillation Mode. *Methods in enzymology* **1997**, *276*, 307-326.

[13] Liebschner, D.; Afonine, P. V.; Baker, M. L.; Bunkoczi, G.; Chen, V. B.; Croll, T. I.; Hintze, B.; Hung, L. W.; Jain, S.; McCoy, A. J.; Moriarty, N. W.; Oeffner, R. D.; Poon, B. K.; Prisant, M. G.; Read, R. J.; Richardson, J. S.; Richardson, D. C.; Sammito, M. D.; Sobolev, O. V.; Stockwell, D. H.; Terwilliger, T. C.; Urzhumtsev, A. G.; Videau, L. L.; Williams, C. J.; Adams, P. D., Macromolecular structure determination using X-rays, neutrons and electrons: recent developments in Phenix. *Acta Crystallogr. D Struct. Biol.* **2019**, *75*, 861-877.

[14] Emsley, P.; Cowtan, K., Coot: model-building tools for molecular graphics. *Acta Crystallogr. D Biol. Crystallogr.* **2004**, *60*, 2126-2132.

[15] Schrödinger Release 2020-1: Maestro, Schrödinger, LLC, New York, NY, 2020

[16] Shivakumar, D.; Williams, J.; Wu, Y.; Damm, W.; Shelley, J.; Sherman, W., Prediction of Absolute Solvation Free Energies using Molecular Dynamics Free Energy Perturbation and the OPLS Force Field. *J. Chem. Theory Comput.* **2010**, *6*, 1509–1519.

[17] Kaminski, G. A.; Friesner, R. A.; Tirado-Rives, J.; Jorgensen, W. L., Evaluation and Reparametrization of the OPLS-AA Force Field for Proteins via Comparison with Accurate Quantum Chemical Calculations on Peptides. *J. Phys. Chem. B* **2001**, *105*, 6474-6487.
